# Disentangling the Relationship Between Chronic Kidney Disease and Cognitive Disorders

**DOI:** 10.3389/fneur.2022.830064

**Published:** 2022-02-25

**Authors:** Dearbhla M. Kelly, Peter M. Rothwell

**Affiliations:** ^1^J. Philip Kistler Stroke Research Center, Department of Neurology, Massachusetts General Hospital, Harvard Medical School, Boston, MA, United States; ^2^Wolfson Center for Prevention of Stroke and Dementia, Nuffield Department of Clinical Neurosciences, John Radcliffe Hospital, University of Oxford, Oxford, United Kingdom

**Keywords:** CKD, dialysis, hypertension, cognitive impairment, dementia, stroke

## Abstract

Chronic kidney disease (CKD) is a rapidly rising global health burden that affects nearly 40% of older adults. Epidemiologic data suggest that individuals at all stages of chronic kidney disease (CKD) have a higher risk of developing cognitive disorders and dementia, and thus represent a vulnerable population. It is currently unknown to what extent this risk may be attributable to a clustering of traditional risk factors such as hypertension and diabetes mellitus leading to a high prevalence of both symptomatic and subclinical ischaemic cerebrovascular lesions, or whether other potential mechanisms, including direct neuronal injury by uraemic toxins or dialysis-specific factors could also be involved. These knowledge gaps may lead to suboptimal prevention and treatment strategies being implemented in this group. In this review, we explore the mechanisms of susceptibility and risk in the relationship between CKD and cognitive disorders.

## Introduction

The global burden of chronic kidney disease (CKD) is rising with estimated prevalence rates of 11–13% (1), increasing to nearly 40% in persons aged 60+ years (2). Although its contribution to cardiovascular diseases is well-established (3), the significant impact of CKD on cognitive brain health is only beginning to emerge. CKD is strongly associated with both cognitive impairment and dementia, and these associations worsen with declining renal function (4). In this review, we will discuss the clustering of risk factors associated with dementia in this group as well as the potential role of novel renal-specific factors. We will endeavor to tease out the role of these putative risk factors and mechanisms as mediators, confounders, or epiphenomena.

## Kidney-Brain Axis

The kidney-brain axis refers to a relationship that exists under both physiological and pathophysiological circumstances. This relationship has been described as the “neglected kidney-brain axis” (5) because the critical interplay between these two organs that can lead to important neurological disease pathophysiology has only recently been recognized. The kidney and brain share similar anatomical and physiological features that render them vulnerable to the impact of traditional cardiovascular risk factors such as hypertension, diabetes, and smoking (6). Both organs share a low vascular resistance system, allowing continuous high-volume perfusion (7). Autoregulation allows constant blood flow despite fluctuations in blood pressure, to maintain cerebral perfusion pressure in the brain and GFR in the kidney. The “strain vessel hypothesis” has been proposed as a possible mechanism for the relationship between renal and cerebrovascular diseases whereby juxtamedullary afferent arterioles in the kidney and cerebral perforating arteries are both exposed to high pressure and have to maintain large pressure gradients, rendering them uniquely susceptible to hypertensive injury (8). This hypertensive vascular injury is then clinically manifest as proteinuria and progressive GFR decline in the kidney, and as symptomatic stroke, silent cerebral small vessel disease and cognitive decline in the brain.

It has also been hypothesized that there may be inflammatory cross-talk between the two organs that may also contribute to the cerebrovascular and neuropsychiatric disease burden observed in patients with CKD (9). This cross-talk between the kidney and brain may include enhanced cytokine/chemokine release and production of reactive oxygen species (ROS) in AKI or CKD leading to neuroinflammation, cytokine interaction with pathogenic neurotrophic factors through a disrupted blood-brain barrier, and activation of the brain renin–angiotensin system (RAS) contributing to oxidative stress via angiotensin II. The cytokines/chemokine release in CKD activates immune cells, neurons, and glial cells in the brain creating a cascade with release of more inflammatory molecules, which locally interact with neurotrophic factors and with ROS, thus contributing to neuropsychiatric disorders.

## Epidemiology of Cognitive Disorders in CKD

The prevalence of MCI in pre-dialysis CKD is reported as variably being between 25 and 62% (10, 11), compared to rates of 11–26% in the matched general population (10, 12). In the Reasons for Geographic and Racial Differences in Stroke (REGARDS) Study, each 10 mL/min/1.73 m^2^ decrease in eGFR <60 mL/min/ 1.73 m^2^ was associated with an 11% increase in prevalence of cognitive dysfunction (13). Haemodialysis patients are three times more likely to have severe cognitive impairment than age-matched non-dialysis patients with reported prevalence rates of 30–40% (14).

CKD is in fact one of the strongest risk factors for mild cognitive impairment (MCI) and dementia as demonstrated by a recent 6-year population-based longitudinal study in which the impact of CKD on risk of MCI and dementia was exceeded only by stroke and chronic use of anxiolytics (15). Even early stages of CKD are associated with cognitive impairment (16). In a pediatric study of 340 patients (ages 6–21) with mild–moderate CKD, a longer duration of CKD was associated with reduced attention and executive function, with a doubling of the odds of poor performance for every 4.6 years of disease exposure (17). However, in the Three-City (3C) Study, a longitudinal cohort of 9,294 adults aged 65 years and over, although the cross-sectional findings suggested that duration of disease was more relevant than the level of GFR; in the longitudinal analysis, rapid eGFR decline (>4 ml/min/1.73m^2^/yr) was more strongly associated with cognitive decline and incident dementia (18). It may be the case that duration of CKD is particularly relevant in children and adolescents during periods of critical neurodevelopment (19).

In another recent, large population-based study, CKD was associated with a higher dementia risk [hazard ratio (HR), 1.71; 95% confidence interval (CI)], 1.54–1.91 in eGFR 30–59 ml/min and HR 2.62, 1.91–3.58 in eGFR <30 ml/min] compared with eGFR of 90–104 ml/min (20). In this study, both the severity of CKD and steeper kidney function decline were associated with dementia. It was found that as many as 10% (95% CI 6–14%) of dementia cases could be attributed to CKD, a proportion higher than that attributed to other dementia risk factors such as cardiovascular disease and diabetes.

As a measure of kidney function, proteinuria also appears to be more strongly associated with cognitive decline than low eGFR for reasons that are unclear (21, 22). This finding is however consistent with recently published meta-analyses data on the relationship between low eGFR, proteinuria, and stroke risk (23, 24).

The prevalence of dementia among haemodialysis patients is 8–37% with the risk increasing linearly with age (12, 25). Prevalence rates are broadly similar (4–33%) for patients on maintenance peritoneal dialysis (12, 26) but fall for kidney transplant recipients (7–22%) (27, 28). Although there may be a selection bias in terms of transplant candidates, improvements in cognitive scores in parallel with favorable structural and functional changes in white matter integrity have been described 1 year after kidney transplantation (29). However, these changes may not be sustained in frail recipients (27). Older adults on haemodialysis with a diagnosis of Alzheimer's disease or dementia have a >2-fold risk of mortality compared to those without these diagnoses (25).

## Mechanisms of Susceptibility and Risk

**Figure 1:**
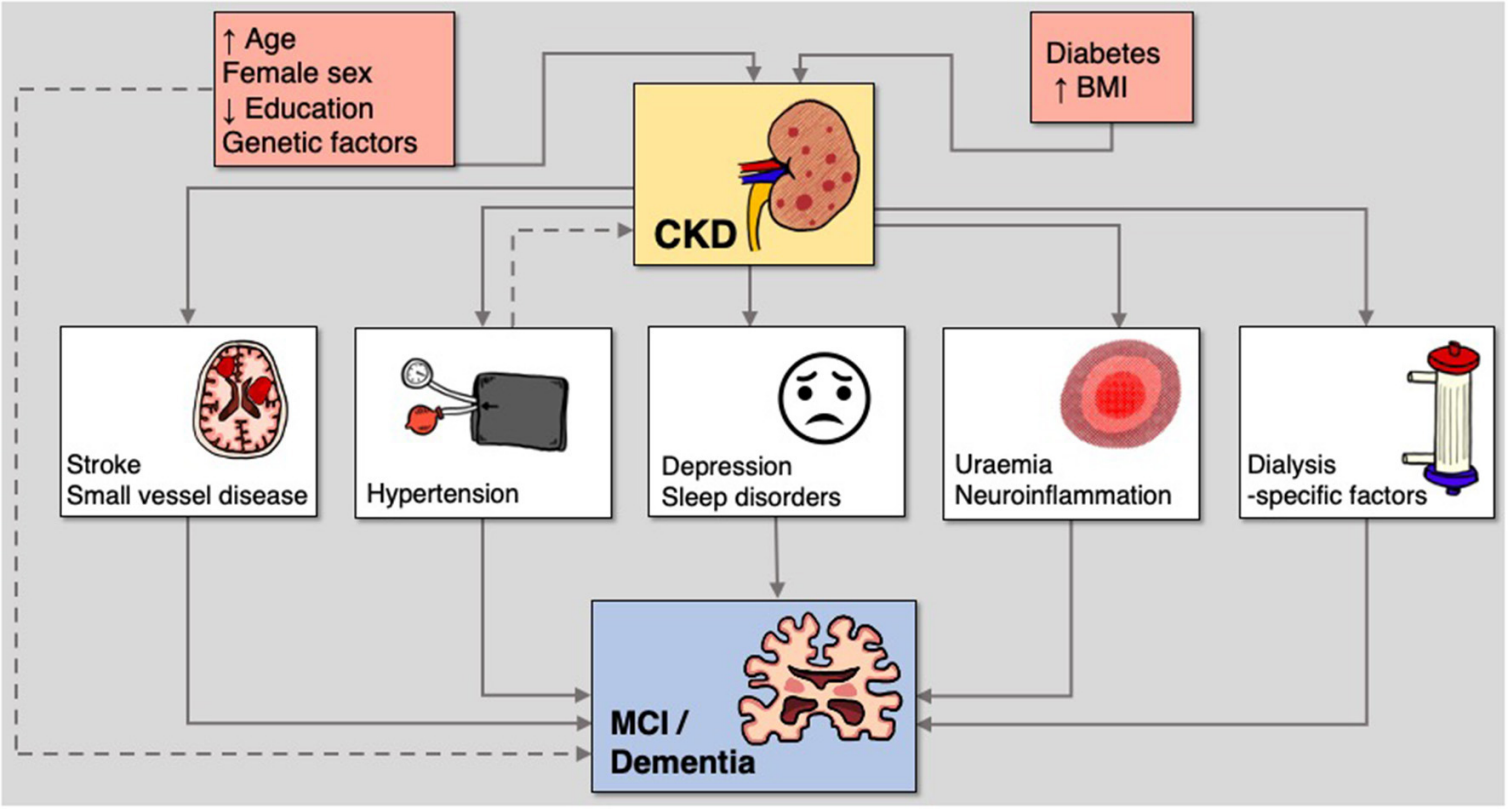
Mechanisms of susceptibility and risk in the relationship between CKD and cognitive disorders.

Mechanisms underlying the pathogenesis of MCI and dementia in CKD are poorly understood. Both vascular and neurodegenerative hypotheses have been proposed (Figure 1) (5, 30). In support of the vascular hypothesis, there is a high prevalence of cardiovascular risk factors, such as hypertension and diabetes mellitus, as well as a significant burden of both symptomatic and subclinical cerebrovascular disease (31). On the contrary, consistent with the neurodegenerative hypothesis, the accumulation of uraemic toxins can cause cerebral endothelial dysfunction and has been implicated in cognitive decline (32). However, this binary view of potential pathogenesis for CKD-related neurocognitive disorders is likely an over-simplistic summary of a multi-factorial process that likely includes elements of both hypotheses. We will outline the evidence for these cognitive risk factors, some of which are shared by the general population, and some of which are renal-specific.

### Age and Sex Differences

The greatest risk factor for Alzheimer's disease (AD) is advanced age (33). Prevalence of AD shows a steep increase with age, from 0.6% in the group age 65–69 years to 22.2% in the group aged 90 years and older (34). Age also contributes to the etiology and progression of CKD. The aged kidney undergoes a range of structural and functional changes that can lead to disordered inflammation and renal fibrosis, rendering the kidney vulnerable to acute insults and increasing the risk of CKD progression (35). These changes may be part of a broader process of systemic persistent inflammation causing inflammatory aging known as “inflammageing”. This condition is characterized by elevated levels of blood inflammatory markers (36), a high susceptibility to cerebrovascular disease and dementia (37, 38), and is exacerbated by uraemia and dialysis dependency (39).

There are also key sex differences in the prevalence of both dementia and CKD. A European meta-analysis found that the pooled prevalence of AD was 7.02 per 1,000 person-years in men and 13.25 per 1,000 person-years in women (40). Women account for approximately two-thirds of patients with AD and related dementias in both Europe and the US (41, 42). This disparity is thought to be attributable to women's greater longevity since risk of developing dementia increases with age and there may be a competing mortality risk for men that can confound HR estimation of dementia (43, 44). However, a recent study showed that incident midlife hypertension was associated with greater memory decline in women and suggested that such discrepancies in risk factor-disease associations could also potentially contribute to heterogeneity of AD disease prevalence in later life (45). Similarly, several other key vascular risk factors such as hyperlipidaemia, diabetes mellitus and atrial fibrillation also appear to be associated with greater risk of stroke in women compared to men which may contribute to downstream dementia burden (46). The proportion of women with pre-dialysis CKD is also higher than that of men, a difference that is also likely accounted for by the longer life expectancy of women, but nonetheless renders them especially vulnerable to accelerated “inflammageing” and the enhanced effects of vascular risk factors, and consequently, to diseases of brain aging such as stroke and dementia (47). Therefore, both age and sex could account for confounding and epiphenomenal association in the relationship between CKD and cognitive impairment.

### Education Level

A low educational level is associated with an increased incidence of clinical AD or dementia (48). It has been suggested that education could delay the clinical expression of dementia symptoms by increasing the neocortical synaptic density (the “brain reserve” hypothesis) (49). Others have proposed that educational and occupational attainment provide a reserve against dementia, enabling this group to cope with advanced pathological changes of the disease more effectively by maintaining function longer (the “cognitive reserve” hypothesis) (50). However, it may also be the case that those with greater educational attainment and associated higher socioeconomic status may be exposed to fewer neurotoxins and have fewer cardiovascular risk factors that may contribute to vascular/neurodegenerative brain disorders (the “brain battering” hypothesis) (51).

Similarly, low educational and occupational levels have been associated with CKD and worse kidney outcomes (52). CKD risk, albuminuria, and reduced eGFR rates are all higher among participants with low educational level compared to those with high educational level. Exploratory longitudinal mediation analysis suggest that the association between education and CKD can partly be explained by diabetes and the modifiable risk factors, body mass index (BMI), waist-to-hip ratio (WHR), smoking, potassium and hypertension (53). Thus, low educational attainment is another potential confounder in the association between CKD and cognitive disorders with some evidence of synergy as subtle GFR decline is associated with more rapid cognitive decline in those with lower educational levels (54). However, more recent data in the general population suggests that higher cognitive reserve may not diminish the adverse effects of covert vascular brain injury (55).

### Hypertension

The causal relationships between hypertension, CKD and dementia are particularly complex as hypertension could be potentially both a confounder and mediator in the relationship between CKD and dementia.

Many observational studies report hypertension to be an important risk factor for dementia (56–58) and in a recent meta-analysis of randomized clinical trials, blood pressure lowering with antihypertensive agents compared with control was significantly associated with a lower risk of incident dementia or CI (59). The relationship between hypertension and cognitive decline may be mediated through cerebrovascular disease (60, 61) or via augmentation of neurodegenerative mechanisms. At autopsy, hypertensive older adults also have evidence of greater AD pathology in the brain, including neurofibrillary tangles and neuritic amyloid-beta (Aβ) plaques (62). Positron emission tomography studies have shown that the extent of Aβ deposition in the brain is positively associated with higher BP (63). The chronicity of past hypertension appears to be most important. Multiple studies have indicated that it is the occurrence of midlife hypertension and its persistence into late life that is one of the leading risk factors for late-life dementia (64, 65).

It follows then that since hypertension occurs in 67–92% of patients with CKD (66), that the adverse cognitive consequences could be accentuated in this group. However, although premorbid mid-life to late-life blood pressure is strongly associated with MCI and dementia in the general population, its role in dementia pathogenesis in CKD is unknown. A recent systematic review and meta-analysis of stroke risk in CKD suggested that most of the risk in this setting may be attributable to long-term blood pressure burden (23). Premorbid blood pressure may therefore also play a similarly central role in the etiology of cognitive dysfunction in CKD, though this has not been previously shown. In an analysis of 8,563 hypertensive adults in the SPRINT trial, they found that a ≥30% decline in baseline eGFR and incident eGFR <60 ml/min/1.73 m^2^ were associated with an increased incidence of probable dementia and MCI, independent of the intensity of hypertension treatment (67). This highlights a potential synergy between hypertension and kidney disease in the pathogenesis of CI and dementia.

### Stroke

Stroke is associated with an increased risk of subsequent dementia. In a large meta-analysis of symptomatic stroke patients, 10% of patients had dementia before first stroke, 10% developed new dementia soon after first stroke, and more than a third had dementia after recurrent stroke (68).

There are also strong associations reported between CKD and cerebrovascular disease (30). Meta-analyses of cohort studies and trials indicate that reduced GFR is associated with a 40% greater risk of stroke and that proteinuria is associated with a 70% greater risk (69) even after adjusting for traditional cardiovascular risk factors. In terms of potential mechanisms, there is a high prevalence of shared vascular risk factors including hypertension, diabetes mellitus, and atrial fibrillation but “non-traditional” risk factors such as anemia, hyperuricemia, and mineral-bone disorders may also play a role (70).

Importantly, several of the predictors of post-stroke dementia (68) are common in the CKD population including older age (35), low educational attainment (52), premorbid disability, (71) and vascular risk factors such as diabetes mellitus and atrial fibrillation (AF) (72). In addition, CKD is associated with several stroke-specific factors (68) that are predictive of post-stroke dementia including higher stroke severity and greater risk of recurrence (73).

### Small Vessel Disease

Cerebral small vessel disease (SVD) is a major etiologic factor in dementia (74). This may relate to a reduction in cerebral blood flow (75), and impaired cerebral autoregulation (76). SVD and AD pathology are thought to interact in important ways (77). Chronic cerebral inflammation due to vascular risk factors exposure and genetic modulators (apoE4) may lead to increase Aβ production while chronic SVD (arteriosclerosis, cerebral amyloid angiopathy) and vascular inflammation may drive inefficient perivascular and cell-mediated Aβ clearance (78).

SVD is highly prevalent in patients with CKD (79) and it is associated with all subtypes including white matter lesions (WML) (80), silent cerebral infarctions (SCI) (81), perivascular spaces (PVS) (82), and cerebral microbleeds (CMB) (83). Over half of all CKD or dialysis-dependent patients have evidence of SCI on imaging studies (84, 85). These associations may relate to the “strain vessel hypothesis” (8), shared cardiovascular risk factor burden (81), or perhaps genetic pleiotropy may play a role in younger populations (86). SCI in the presence of CKD has been associated with executive dysfunction (87). This pattern of cognitive change with prominent impairment of executive function and processing speed has also been observed in maintenance haemodialysis patients (88), consistent with cognitive deficits associated with cerebrovascular disease (89). It is therefore unclear whether CKD is a risk factor for dementia independent of either symptomatic or subclinical cerebrovascular disease.

### Diabetes Mellitus and Obesity

A recent meta-analysis of over 2 million participants showed that individuals with type 2 diabetes are at ~60% greater risk for the development of dementia compared with those without diabetes (90). Those with a younger age of diabetes onset and cardiovascular comorbidity are particularly at risk (91). Several mechanisms for the link between diabetes and dementia have been proposed including brain metabolic dysfunction as a driver for AD pathology (92), with impairments in insulin transport through the blood-brain barrier, insulin signaling, and resultant decreased cerebral glucose utilization (93). In addition, hyperglycemia may lead to neurotoxicity, vascular injury, and accumulation of advanced glycation end products (94). Nearly one third of CKD is attributable to diabetic nephropathy (3) and even patients with mild-moderate stages of diabetic kidney disease have been found to have occult neurocognitive disorders (95), highlighting the role of diabetes as a potential confounding factor in this pathway.

Increasing evidence suggests that obesity, highly prevalent in the CKD population (96) and estimated to account for ~20–25% of kidney disease worldwide (97), is also an independent risk factor for dementia. In an analysis of 6,582 participants from the English Longitudinal Study of Aging, individuals with baseline obesity had about a 30% increased risk of dementia even after adjusting for sex, baseline age, apolipoprotein E-ε4 (APOE-ε4), education, physical activity, smoking, marital status, hypertension and diabetes (98). Similar to diabetes though, excess adiposity is linked with a change in brain energy metabolism, the accumulation of brain lesions and brain volume loss leading to neurodegeneration (99).

### Depression and Sleep Disorders

Approximately 25% of CKD patients report symptoms of a major depressive disorder (100) with high rates of under-treatment described (101). In particular, hemodialysis patients with a greater burden of depressive symptoms perform worse on tests of cognition related to processing speed and executive function, suggesting that depression could therefore be a potential mediating or contributing factor in the relationship between CKD and cognitive disorders (102).

Similarly, sleep disorders are highly prevalent in CKD with a spectrum of manifestations described including insomnia, sleep fragmentation, daytime somnolence, sleep apnoea, altered circadian rhythm, and restless legs syndrome (103). Sleep disorders are also highly linked to cognitive impairment and dementia and are often representative of underlying brain pathology (104). The glymphatic system is responsible for clearance of ~60% of β-amyloid clearance and since this occurs primarily during sleep (105), which is altered during CKD, it has been proposed that glymphatic fluid transport may be suppressed in CKD, leading to an accumulation of potentially neurotoxic waste products (106).

### Genetic Factors

The role of genetic factors in the pathogenesis of cognitive dysfunction in CKD has been largely unexplored (106). In younger patients, some rare genetic syndromes have been described that can cause both kidney disease as well as neurocognitive disorders including tuberous sclerosis (107), Fabry disease (108), and Bardet-Biedl Syndrome (109). In general, compared with noncarriers, children with genetic kidney disease score significantly poorer on all measures of intelligence, anxiety/depressive symptoms, and executive function (110).

A genetic cause has been described in 10% of adult patients with CKD (111), and this figure can rise to 37% of those with positive family history, many of whom have extra-renal features (112). However, it is not known whether there is a similar tendency toward neurocognitive disorders in this group. Several single-nucleotide polymorphisms (SNPs) associated with kidney disease (113) are in exons for genes that also expressed in the brain including in the striatum (SLC47A1, KLHDC7A and SLC25A45; from the Allen Brain Atlas database), cortex (EDEM3, PPM1J, and CERS2; from the Human Protein Atlas database) and the cerebellum and hippocampus (TSPAN9 and EPB41L5; from the Human Protein Atlas database). Furthermore, some are in genes linked to Alzheimer's disease (CACNA1S; WikiPathways database).

Two genome-wide association studies have also previously indicated genetic pleiotropy between kidney and cerebrovascular disease, particularly with large artery atherosclerotic and small vessel stroke (86, 114). In the most recent of these studies that leveraged large-scale data from international consortia, a locus at 2q33 showed pairwise associations between urinary albumin:creatinine ratio and both small vessel stroke and white matter hyperintensities (WMH), indicating that 2q33 may play a role across small vessel pathologies in both the kidney and brain through microalbuminuria, small vessel stroke, and WMH, and that there may be a shared common pathway among cerebral and renal manifestations of small vessel disease (114).

### Uraemia and Neuroinflammation

The accumulation of uraemic toxins is proposed to cause cerebral endothelial dysfunction and contribute to cognitive disorders in CKD (32). High uraemic toxin concentrations of guanidine compounds such as creatinine, guanidine, guanidinosuccinic acid, and methylguanidine have been found in CKD patients in strategic brain regions for cognition, such as the thalamus, mammillary bodies, and cerebral cortex (115). Haemodialysis efficiently eliminates water-soluble toxins and improves acute uraemic encephalopathy, but is relatively ineffective for protein-bound or medium-sized toxins that may contribute to chronic cognitive dysfunction in patients with ESKD (106). Of particular interest is Neuropeptide Y, a polypeptide that has been implicated in some neurodegenerative and neuroimmune disorders (116), and that is also present in high levels in CKD (117).

Inflammation has also been suggested as a mediator of cognitive decline in CKD (118). The intensity of systemic inflammation, as indicated by elevations in multiple markers of inflammation, including interleukin-1β (IL-1β), interleukin-6 (IL-6), tumor necrosis factor–α (TNF-α), and C-reactive protein (CRP), appears to increase as kidney function declines (119). Both cross-sectional and longitudinal studies have shown that that CRP and fibrinogen are independently associated with deterioration in some domains of cognitive function in patients with CKD (120, 121), though these studies are vulnerable to type 1 error from multiple testing.

### Dialysis-Specific Factors

**Figure 2:**
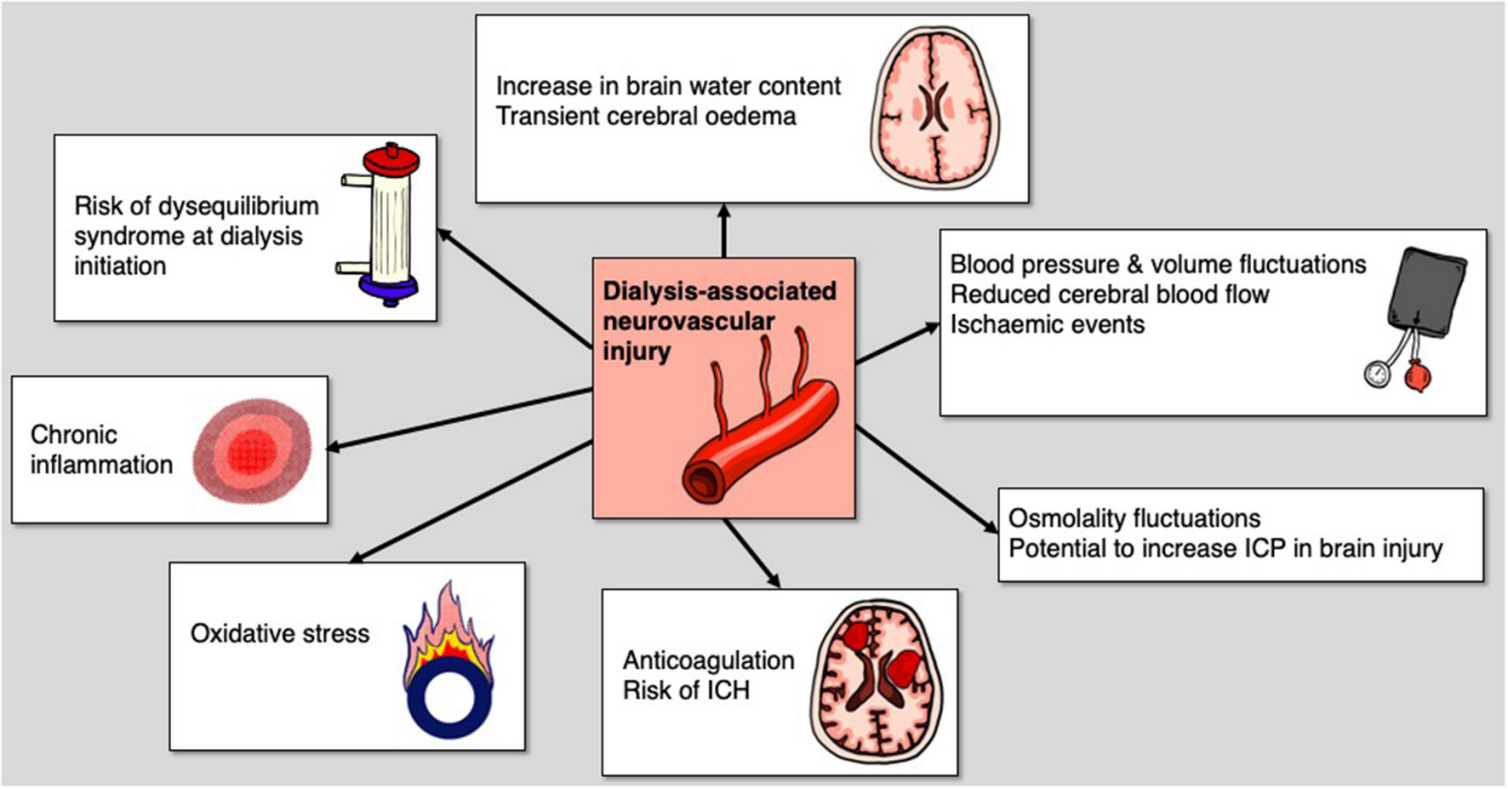
The potential impact of dialysis-associated neurovascular injury on cognition.

It is increasingly recognized that haemodialysis is associated with both acute and chronic brain injury (122, 123). Even in clinically stable patients undergoing intermittent haemodialysis, it can cause cerebral oedema via an increase in brain water content and from reverse osmotic shift due to urea (124) or other newly formed brain osmoles (125). Global cerebral blood flow has also been shown to decline acutely by 10% during hemodialysis (126). Thus, in the setting of acute brain injury, there is a risk of secondary brain injury in what's now referred to as dialysis-associated neurovascular injury (DANI) (Figure 2) (122).

In the chronic setting, it has been shown that every 10 mmHg drop from baseline in mean arterial pressure during a dialysis session is associated with a 3% increase in ischaemic events (127). Nearly one-quarter of haemodialysis sessions feature cerebral ischaemic events and these intradialytic events correlate with decreased executive cognitive function at 12 months.

In a prospective cohort study of about 100 chronic haemodialysis patients, cerebral arterial mean flow velocity (MFV) was demonstrated to decline significantly during dialysis and this decline correlated with intradialytic decline in cognitive function (128). Decline in MFV also correlated significantly with progression of white matter burden and cerebrovascular disease at 12 months follow-up. Haemodialysis is thus capable of inducing transient “cerebral stunning,” analogous to myocardial stunning, and may be a major mechanism of cerebral injury and accelerated cognitive decline in dialysis-dependent patients.

### Beta-Amyloid Pathology

The role of beta-amyloid (Aβ) pathology in the relationship between CKD and cognitive decline in poorly understood. Serum Aβ levels have been shown to be significantly higher in CKD patients, possibly related to reduced renal clearance of Aβ protein from peripheral blood (129). Cystatin-C, a low-molecular weight protein that is used to estimate GFR, has also been demonstrated to colocalize with beta-amyloid in the brain (130).

However, there is some evidence from animal and small human studies that peripheral clearance of Aβ by dialysis could help to reduce the amyloid plaque burden in the brain (131). In one study, plasma Aβ levels before and immediately after peritoneal dialysis in 30 patients with newly diagnosed CKD and in APP/PS1 mice were measured. In both cases, plasma Aβ40 and Aβ42 levels were significantly reduced after dialysis. In the animal model, PD resulted in a decrease in Aβ levels in the brain interstitial fluid with reduced plaque deposition. Dialysis solution appeared to account for only 10% of Aβ removal suggesting that the remaining clearance was mediated by efflux transport of Aβ across the BBB and enhancement of endogenous clearance pathways. The dialysis-treated mice showed reduced levels of hyperphosphorylated tau in the brain, suggesting a slowing of neurodegeneration along with decreased inflammation. Attenuated cognitive decline was demonstrated by improved performance on the Y-maze and open-field tests.

Brain Aβ deposition also appears to be lower in maintenance haemodialysis patients (132). Clearance rates of both peptides during one haemodialysis session were 22% and 35% for Aβ42 and Aβ40, respectively (133). By inducing peripheral Aβ sink and stimulating Aβ efflux from the brain, it has been suggested that haemodialysis could be considered as an anti-amyloid treatment strategy.

## Conclusions

CKD is strongly associated with MCI and dementia, and the pathogenesis is likely multifactorial, incorporating elements of both vascular disease as well as neurodegenerative processes. Patients with CKD appear to have a clustering of susceptibility and risk factors associated with dementia including lower cognitive reserve (advancing age, lower educational and occupational attainment), cardiometabolic risk factors (hypertension, diabetes, obesity, stroke), neuropsychiatric comorbidities (depression, sleep disorders) and renal-specific factors (uraemia, inflammation, intradialytic “cerebral stunning”). From an epidemiological perspective, it remains challenging to disentangle independently causal associations from intermediate mediators, confounders, and epiphenomena. Further research is needed to fully elucidate the role of genetic factors and Aβ pathology in this relationship. In an aging population, targeting novel modifiable risk factors such as CKD and associated multimorbidity may help reduce the global burden of dementia.

## Author Contributions

DK drafted the manuscript for intellectual content. PR contributed to the format and revised the manuscript for intellectual content. Both authors contributed to the article and approved the submitted version.

## Funding

DK is an Atlantic Fellow for Equity in Brain Health at the Global Brain Health Institute (GBHI) and is supported with funding from GBHI, Alzheimer's Association, and Alzheimer's Society (GBHI ALZ UK-22-868940). DK is also the recipient of an NIH StrokeNet Fellowship. PR has received funding from Wellcome Trust, Wolfson Foundation, British Heart Foundation, National Institute for Health Research, and the National Institute for Health Research Oxford Biomedical Research Center.

## Conflict of Interest

The authors declare that the research was conducted in the absence of any commercial or financial relationships that could be construed as a potential conflict of interest.

## Publisher's Note

All claims expressed in this article are solely those of the authors and do not necessarily represent those of their affiliated organizations, or those of the publisher, the editors and the reviewers. Any product that may be evaluated in this article, or claim that may be made by its manufacturer, is not guaranteed or endorsed by the publisher.
